# Impact of a non-constant baseline hazard on detection of time-dependent treatment effects: a simulation study

**DOI:** 10.1186/s12874-021-01372-0

**Published:** 2021-08-28

**Authors:** Kim Jachno, Stephane Heritier, Rory Wolfe

**Affiliations:** School of Public Health and Preventive Medicine, Monash University, Melbourne, Victoria Australia

**Keywords:** Non-proportionality, Non-constant hazards, Flexible parametric models, Weighted logrank tests, Restricted mean survival time

## Abstract

**Background:**

Non-proportional hazards are common with time-to-event data but the majority of randomised clinical trials (RCTs) are designed and analysed using approaches which assume the treatment effect follows proportional hazards (PH). Recent advances in oncology treatments have identified two forms of non-PH of particular importance - a time lag until treatment becomes effective, and an early effect of treatment that ceases after a period of time. In sample size calculations for treatment effects on time-to-event outcomes where information is based on the number of events rather than the number of participants, there is crucial importance in correct specification of the baseline hazard rate amongst other considerations. Under PH, the shape of the baseline hazard has no effect on the resultant power and magnitude of treatment effects using standard analytical approaches. However, in a non-PH context the appropriateness of analytical approaches can depend on the shape of the underlying hazard.

**Methods:**

A simulation study was undertaken to assess the impact of clinically plausible non-constant baseline hazard rates on the power, magnitude and coverage of commonly utilized regression-based measures of treatment effect and tests of survival curve difference for these two forms of non-PH used in RCTs with time-to-event outcomes.

**Results:**

In the presence of even mild departures from PH, the power, average treatment effect size and coverage were adversely affected. Depending on the nature of the non-proportionality, non-constant event rates could further exacerbate or somewhat ameliorate the losses in power, treatment effect magnitude and coverage observed. No single summary measure of treatment effect was able to adequately describe the full extent of a potentially time-limited treatment benefit whilst maintaining power at nominal levels.

**Conclusions:**

Our results show the increased importance of considering plausible potentially non-constant event rates when non-proportionality of treatment effects could be anticipated. In planning clinical trials with the potential for non-PH, even modest departures from an assumed constant baseline hazard could appreciably impact the power to detect treatment effects depending on the nature of the non-PH. Comprehensive analysis plans may be required to accommodate the description of time-dependent treatment effects.

**Supplementary Information:**

The online version contains supplementary material available at 10.1186/s12874-021-01372-0.

## Background

Randomised clinical trials (RCTs) have an overarching objective to understand if a new treatment is effective compared to existing treatments. RCTs with time-to-event outcomes can examine when, and for how long, the treatment exhibits an effect. Nevertheless, the vast majority of RCTs with time-to-event outcomes are analysed using methods that are maximally powerful under an assumption of proportional hazards, implying time-independent or ‘fixed’ magnitude treatment effects. The main analytical approaches currently reported in major medical journals can be broadly categorised as tests of equal survival functions which provide a *p*-value for inference only, or modelling approaches which provide an estimate of treatment effect along with a *p*-value for inference [1–5]. When designing trials, as well as the assumption of time-independent treatment effects, there is often an explicit or implicit assumption of constant event rates – constant baseline hazards - used to determine the number of events required and hence the number of patients that need to be recruited for the trial to have the desired power in the sample size calculations methods employed [4, 6].

Paradigm shifts in oncology treatments over the past two decades provides motivation for assessing the effect of non-proportionality on analytical methods for time-to-event outcomes [7]. Two broad classes of time-dependent treatment effects, early effect that attenuates and lag to effect, have emerged as there has been a shift to biomolecular-targeted and immunotherapy-based treatments implemented either alone or as an adjunct to surgical and chemotherapy-based approaches. Many of the first wave of biomolecular-based anticancer agents were observed to improve patient survival initially but have limited long-term survival benefit due to acquired biological resistance to, or accumulated toxicities from the treatment. This is an example of an early treatment effectiveness which attenuates or becomes harmful over time. A subsequent wave of immunotherapy-based treatments act to stimulate the patient’s own immune system to kill cancerous cells. This circumvents the problems observed with toxicity and resistance to the biological-based agents. However, this mechanism of action via immune system activation is typically associated with a delay of varying months’ duration until any treatment effect may be observed, an example of a lag until treatment effectiveness. Recent reappraisals using reconstructed data of published phase III oncology trials have highlighted how prevalent time-dependent treatment effects may be, and that the use of standard analytical approaches assuming time-fixed treatment effects may underestimate the magnitude of, or miss completely. Treatment effects that provide substantial survival benefits [5, 8].

The two most popular analysis approaches for comparing survival curves in different treatment groups are the logrank (LR) test used to evaluate the null hypothesis of identical survival functions, and the Cox PH model to obtain an estimate of the treatment effect as a summary hazard ratio (HR). Under PH, these two approaches are known to be maximally powerful and provide an asymptotically equivalent test of significance. When non-proportionality exists, the LR test can lose power to detect survival curve differences with the magnitude of the loss dependent on the configuration of the non-proportionality. Extensions to the LR test have been proposed which maintain power under different anticipated scenarios of non-proportionality. These include the Fleming-Harrington (FH) family of weighted LR test statistics which can be differentially weighted to emphasise events that occur earlier, in the middle, or later over the survival time horizon of interest [9]. Other weighting approaches exist that use more flexible data-driven procedures to specify weight functions that maintain power, such as Yang and Prentice’s adaptive model [10] or Magirr and Burman’s modestly weighted LR test for delayed-onset non-proportionality [11]. Weighted LR tests can be criticised because they treat some events as more important than others and that there is not necessarily an accompanying estimate of treatment effect available for clinical interpretation. An alternative approach to testing for a generalised treatment effect is to use the combined results of multiple significance tests appropriately standardised to maintain the null distribution. Examples of these combined tests include using the minimum of the Cox PH model *p*-value and a permutation test based on the restricted mean survival time [12] or selecting the minimum of the three *p*-values from the FH family weighted LR tests under equal, early effect and lag to effect weighting scenarios [13].

When the assumption of proportionality of the treatment effect is met, the summary HR from a Cox PH model is a suitable parameter to provide a clinically meaningful measure of the relative difference between two survival curves. When not met, the clinical interpretation of a single summary measure such as the HR is not clear. When the underlying HR varies over time, assuming that there are a series of periods in which the PH assumption holds, then the magnitude of the summary HR can be interpreted as a weighted average of the sum of the proportion of events and estimated HR in each of the periods. These weights depend on the event rates, accrual distribution and the dropout pattern, and these dependencies could result in different parameter estimates in different trials, even with identical survival curves, thus removing the integrity of the summary HR as a meaningful measure of overall treatment effect.

An alternative estimand of treatment effect for time-to-event outcomes that does not rely on the PH assumption is the restricted mean survival time (RMST) [14]. The RMST is the mean duration of survival for the trial population up to a given time point (often designated *t*^∗^). Recent research on the use of the RMST to estimate treatment effects as an adjunct estimand to the HR has shown agreement in terms of statistical significance of the treatment effect under PH [14–16]. Since the choice of estimand and analytical method needs to be pre-specified in a clinical trial, to avoid any bias from selective reporting, a summary HR from a Cox model is often stipulated as the primary analysis because at that point in time there may be an absence of meaningful data from which to justify the treatment effect as a time-varying quantity. However, it has been recommended that the difference in RMST, or the ratio of RMST, be reported complementary to, or as the primary outcome measure in trials whether or not non-proportionality of the treatment effect could be anticipated [17, 18]. As well as not relying on a PH assumption, the RMST also has desirable properties for (i) interpretability in that it can be expressed in both relative and absolute measures and the chosen metric is time, not risk, and (ii) performance since it is a summary measure that captures the temporal profile of all events up to the cut off time *t*^∗^.

When conducting clinical trials, in order for a single test of RMST difference to be valid, the selected time point of interest *t*^∗^ must be pre-specified at the design stage. Choices of *t*^∗^ relatively late in the follow up confer power similar to that observed with the Cox PH model. Depending on the patterns of non-PH, other choices of *t*^∗^ may considerably increase the power to detect a difference. Royston and Parmar have also developed a generalised test of treatment effect, which tests the RMST difference at several prespecified values of *t*^∗^ during the follow-up, taking the smallest *p*-value as the basis for the test after adjusting for multiple testing [12]. By combining this *p*-value and the *p*-value from the Cox PH model, an overall p-value for the combined test (designated pCT) can be derived and has the correct distribution under the null hypothesis of equal survival curves.

Accelerated failure time (AFT) models [19–21] also model the treatment effect on a time-based rather than a hazard-based metric, enabling potentially more intuitive clinical understanding. These models include a survival model based on the Weibull distribution which has both PH and AFT interpretations depending on the parameterisation selected, thus acting as a conduit model for investigating treatment effects in both risk-based and time-based metrics.

A further consideration, as yet unexamined in the comparisons of the performance of analysis methods, is the shape of the hazard in the baseline treatment group. Reviews of adequacy of the event rate parameters used in sample size calculations compared to that observed in the trial have found that event rates were often underestimated [22] or that there were large discrepancies between the assumed parameters and the estimated ones from observed data [23]. Sample size calculations assuming constant, or at the most, piecewise constant event rates were applied even when prior information on the shape of the underlying event rate was available [6].

The Cox model makes no assumption about this shape whereas parametric modelling approaches, including fractional polynomials [24] or splines [25] model the underlying shape of the baseline hazard function. If the PH assumption holds, the time when the events occur does not influence the magnitude, coverage, power or type I error rate of the HR estimate. However, in the presence of a time-dependent effect of treatment, the summary HR provides an ‘average’ effect with the averaging being weighted by the number of events and the timing of their occurrence. While it is reasonably intuitive [14] to infer that the shape of the hazard function in the control group will impact on the extent to which a HR from a Cox PH model is a misleading summary of time-dependent effects of treatment, there is limited work that has quantified this phenomenon nor explored general properties of the Cox PH model HR estimand when the model is mis-specified in this way. The properties of other analytical approaches that estimate effects of treatment have also not been examined in this context.

This paper evaluates the impact of a non-constant event rate on the suitability of three measures of treatment effect - the HR, the difference in RMST (*Δ* RMST), and an acceleration factor expressed as a time ratio (TR) under scenarios where PH do not hold. Suitability of the treatment effect estimates will be assessed in terms of their estimated magnitude, coverage and power benchmarked to that assumed at the design phase of the trial. The properties of three modelling approaches will be examined, the semiparametric Cox PH model, the Royston-Parmar (RP) models utilising flexible restricted cubic splines and parametric models assuming the exponential or Weibull distributions. A landmark (LM) approach to the parametric modelling that allow for multiple estimates of time period-specific or conditional treatment effects will also be undertaken. Additionally, the impact of non-constant event rates on the power of commonly pre-specified analytical approaches that provide a test of equal survival curve significance but not an estimate of treatment effect will be assessed. These approaches include using the *p*-values obtained from the Cox PH model, the LR test, weighted LR tests and omnibus extensions to the weighted LR test and the combination test based on the RMST.

The structure of the article is as follows. In the Methods section we describe the aims of the simulation study, the data-generating models used for the different non-PH scenarios, the estimands of treatment effect and tests of equal survival functions to be compared and the measures used to assess the performance of the analysis methods. In the Results section, we report the results of the findings of the simulations. We end with a Discussion and some recommendations and conclusions.

## Methods

We aimed to assess the effect of non-constant event rates on the suitability of the estimates from three measures of treatment effect, the HR, the time ratio (TR) and the *Δ* RMST, and on the performance of tests of equal survival function under PH and two non-PH scenarios. Our motivation came from phase II and III clinical trials of immunotherapies for late stage cancers [5, 8]. In the absence of treatment, most participants were likely to experience the event of interest within the study’s proposed follow-up time of 50 months. We based the simulation on a generic two-group trial to detect a 33% reduction in the hazard rate underlying progression-free survival with 80% power and a significance level 0.05. Assuming a constant – or equivalently proportional - event rate and PH, a sample size calculation based on the LR test with HR = 0.67, (log (HR) = − 0.4) would require 202 events to be observed [26]. Characteristics of the Design model used in the simulations are detailed in Table 1, along with the Data-Generating models (DGMs) for the simulation and Analysis models that could be chosen for pre-specification in a trial protocol.

**Table 1:** Characteristics of the Design model, the Data-Generating models and the Analysis models

**Design Model:**	Weibull baseline hazard (constant event rate), proportional hazards (PH), treatment effect HR = 0.67, maximum time *t* = 50		
	*h*(*t*) = *λγt*^*γ −* 1^ exp. (*β*X_*TRT*_) where *λ* = 0*.*10*, γ* = 1*.*0*, β* = *−* 0*.*4 and X_*TRT*_ = 0*,*1 for control and treatment groups		
**Data Generating Models (DGMs):**	Weibull baseline hazard (decreasing, constant and increasing event rates), non-proportional hazards		
	Event rate scenario	Baseline hazard values	Non-proportional hazard change times
	**Lag until effect**, HR = 1 if *t ≤ t*_*lag*_, HR = 0.67 if *t > t*_*lag*_; *h*(*t*) = *λγt*^*γ −* 1^ exp. (*β*X_*TRT*_ *× I*(*t > t*_*lag*_))		
	Decreasing	*λ*_*d*_ = 0*.*15*, γ*_*d*_ = 0*.*9	*t*_*lag*_ = 0*,* 1*,* 3 or 10; *t*_*lag*_ = 0 are PH DGMs
	Constant	*λ*_*c*_ = 0*.*10*, γ*_*c*_ = 1*.*0	
	Increasing	*λ*_*i*_ = 0*.*07*, γ*_*i*_ = 1*.*1	
	**Early effect ceasing**, HR = 0.67 if *t ≤ t*_*early*_, HR = 1 if *t > t*_*early*_; *h*(*t*) = *λγt*^*γ −* 1^ exp. (*β*X_*TRT*_ *× I*(*t ≤ t*_*early*_))		
	Decreasing	*λ*_*d*_ = 0*.*15*, γ*_*d*_ = 0*.*9	*t*_*early*_ = 3*,*10*,*20,50; *t*_*early*_ = 50 are PH DGMs
	Constant	*λ*_*c*_ = 0*.*10*, γ*_*c*_ = 1*.*0	
	Increasing	*λ*_*i*_ = 0*.*07*, γ*_*i*_ = 1*.*1	
**Analysis Models:**	Cox PH (Cox)	*h*_*i*_(*t*) = *h*_0_(*t*) exp. (*β*X_*TRT*_)	Average HR from all events in *t*
	Landmark (LM)	*h*_*i*_(*t*) = *h*_0_(*t*) exp. (*β*X_*TRT*_ *× I*(*t > t*_*LM*_))	Average HR from events *after t*_*LM*_^a^
	Piecewise exponential (PE1)	*h*_*i*_(*t*) = *λ*_*j*_ exp. (*β*X_*TRT*_)	Average HR from all events in *t*
	Piecewise exponential (PE2)	*h*_*i*_(*t*) = *λ*_*j*_ exp. (*β*X_*TRT*_ *× I*(*t > t*_*PE*_))	Average HR from events *after t*_*PE*_^b^
	Royston Parmar PH (RP (PH))	ln (*H*_*i*_(*t*)) = *s* (ln(*t*)*|****γ***_*s*_*,***k**_0_) + *β*X_*TRT*_	Average HR from all events in *t*
			∆RMST from all events in *t*
	RP time-dependent (RP (TD))	ln (*H*_*i*_(*t*)) = *s* (ln(*t*)*|****γ***_*s*_*,***k**_0_) + *s* (ln(*t*))*X*_*TRT*_ + *β*X_*TRT*_	∆RMST from all events in *t*
	Accelerated Failure Time (AFT)	ln (*t*_*i*_) = *β*X_*TRT*_ + ε_*i*_	Average TR from all events in *t*

^a^Pre-specified *t*_*LM*_ = 3 for lag until effect non-PH, *t*_*LM*_ = 10 for early effect ceasing non-PH

^b^Pre-specified *t*_*PE*_ = 3 for lag until effect non-PH, not reported for early effect ceasing non-PH

### Data-generating processes for simulation scenarios

**Fig. 1:**
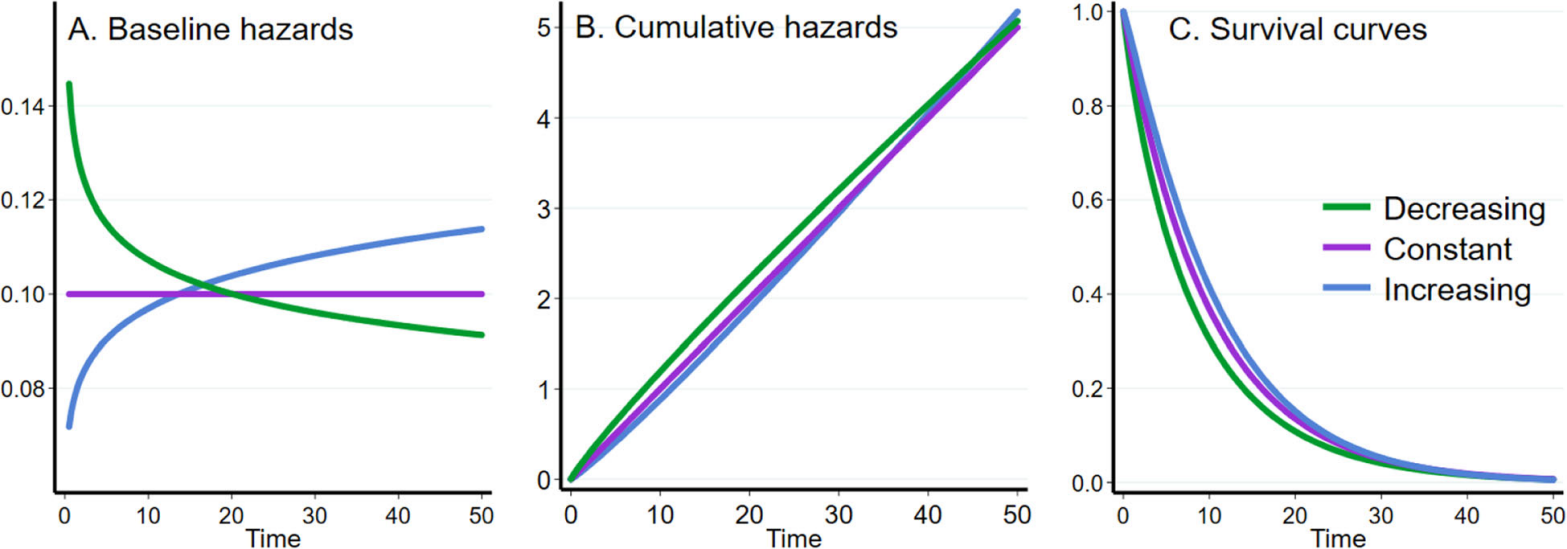
Three event rate scenarios depicted on the hazard scale, cumulative hazard and survival curves. Lines depict baseline hazards – or instantaneous risk of event occurrence in the control group over time – under the three scenarios used for data generation. Decreasing, constant and increasing event rate scenarios are indicated by the green, purple and blue lines respectively. By design, the survival proportion will be the same at t = 50 under all three event rates

Using a Weibull data-generation model, three different event rate scenarios were considered by selecting a scale parameter *λ* and a shape parameter *γ* such that there was a near zero probability of survival by the end of an administratively imposed time in each scenario. For the constant event rate scenario, we determined the value for the scale factor (*λ*_*c*_) that would result in less than 0.7% chance of survival in the absence of treatment effect under a constant event rate (*γ*_*c*_ = 1; ie the exponential distribution) within the specified trial time frame (*t* = 50 months). In the second and third scenarios, clinically plausible values of the shape parameter were selected to provide modest decreasing (*γ*_*d*_ = 0.9) and increasing (*γ*_*i*_ = 1.1) event rate scenarios. For these latter scenarios, we determined the scale parameter that would result in the same survival probability by the end of follow up (*t* = 50), and hence observation of the same number of events in the absence of treatment, as under the constant event rate (see Table 1). This enabled us to assess the effects of non-constant event rates on the different analytical approaches with the same total number of events in each scenario with only the timing of the events differing due to the selected shape of the baseline hazards. We selected modest values of the shape parameter to assess the impact of non-constant event rates in circumstances where an assumption of constant event rates at the design stage of the trial would have been considered appropriate. Use of more extreme values of the shape parameter may have resulted in far more impactful effects on simulation performance measures, but would not have been reflective of typical experiences with clinical trials. The baseline hazard, cumulative hazard and survival functions for the three event rate scenarios for the control and treatment groups are shown in Fig. 1.

**Fig. 2:**
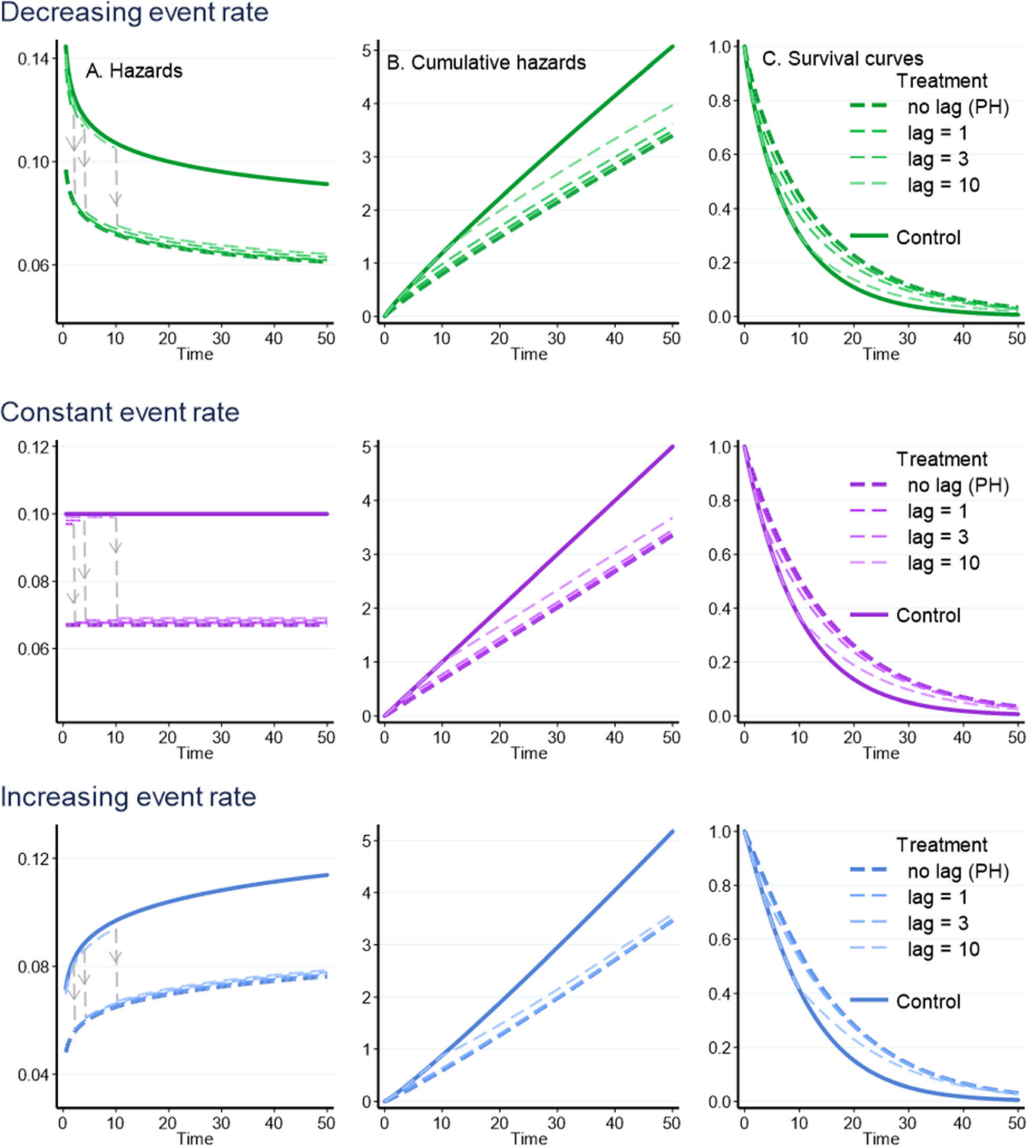
Hazard functions, cumulative hazard curves and survival curves for lag until effect non-PH scenario. Lag period lengths investigated were *t*_*lag*_ = 0, 1, 3 and 10 months within the maximum follow-up time *t* = 50, with the setting *t*_*lag*_ = 0 representing PH. The lag period instantaneous change point times from control group hazard to treatment group hazard are indicated by the vertical gray lines. Decreasing times for treatment effectiveness as a result of increasing lag times are indicated by the decreased shading of the dashed lines used for the treatment group. Decresing, constant and increasing event rate scenarios are indicated by the green, purple and blue lines respectively

Event times were simulated using the survsim command in Stata [27]. A binary covariate for treatment group status (*X*_*trt*_) was simulated from a Bernoulli random variable with probability *p* = 0.5 to mimic 1:1 randomisation. Non-proportional hazards were introduced by dividing the analysis time into two periods with a change point at *t*_*lag*_ or *t*_*early*_ depending on the non-PH scenario. The baseline hazard in the control group was either a decreasing, constant or increasing continuous event rate the same as depicted in Fig. 1A. For simulations investigating a lag until treatment effect, the hazard in the treatment group during the first period prior to *t*_*lag*_ was the same as in the control group, ie there was no effect of treatment (*β* = 0). After *t*_*lag*_ the hazard in the treatment group had the anticipated beneficial design effect (*β* =  − 0.4). The lag period lengths investigated were *t*_*lag*_ = 0, 1, 3 and 10 months within the maximum follow-up time *t* = 50, with the setting *t*_*lag*_ = 0 representing PH. The three lag durations were selected to enable us to investigate a range of power values and treatment effect magnitudes from the stipulated design values to nearly null values, with the maximum delayed effect of 20% of study duration the longest lag time likely to be encountered in practice. The hazard, cumulative hazard and survival functions for the PH and increasing lag until effect times for the control and treatment groups under the decreasing, constant and increasing event rate scenarios are shown in Fig. 2.

Simulations were also performed for the scenario of a treatment that is effective for an initial period then ceases. The period prior to *t*_*early*_ was the period in which the treatment had the anticipated design effect (*β* =  − 0.4), and the period after *t*_*early*_ was when there was no effect of treatment (*β* = 0). The early effect period lengths investigated were *t*_*early*_ = 3, 10, 20 and 50 months, with the setting *t*_*early*_ = 50 representing PH. Again, these early effect durations were selected to cover power values and treatment effect magnitudes from nearly null to the nominal design values. The DGM section of Table 1 details the simulation characteristics for survival data for three different baseline hazard functions under PH and two different non-PH scenarios. Supplementary Figure S1 presents the hazard, cumulative hazard and survival functions for the PH and early effect that ceases non-PH scenarios for the decreasing, constant and increasing event rates in Additional file 1.

### Estimands of treatment effect

The estimands of treatment effect in the simulation study were the hazard ratio, the time ratio and the difference in restricted mean survival time.

#### Hazard ratio (HR)

The HR is obtained by comparing the instantaneous event rates in the treatment group (X_*trt*_ = 1) to the control group (*X*_*trt*_ = 0). For the Weibull data generation model, the effect of treatment is measured as

$$\mathrm{HR}=\frac{\exp \left({\beta}_0+{\beta}_1\right)\gamma {t}^{\gamma -1}}{\exp \left({\beta}_0\right)\gamma {t}^{\gamma -1}}=\exp \left({\beta}_1\right)$$

where *β*_1_ is the co-efficient of the covariate for treatment group status. In the simulation study comparing different modelling approaches, summary estimates of HR were obtained by fitting a Cox PH model, a piecewise exponential (PE) regression model and a Royston-Parmar model [28] under the assumption of PH (time-fixed treatment effects). Time-period specific estimates of HR, either conditional on being event-free at a pre-specified landmark time point, or from allowing an interaction with a discrete-period time point indicator in the PE model were also measured.

#### Difference in restricted mean survival time (Δ RMST)

The RMST *μ* of a time-to-event random variable *T* is the mean of min (*T*, *t*^∗^) where the cut off time *t*^∗^ is greater than zero. RMST can be derived as the area under the survival curve *S*(*t*) = *P*(*T* > *t*) from *t* = 0 to *t* = *t*^∗^. In a randomised two-group trial with survival functions ${S}_{X_T}(t)$ and ${S}_{X_C}(t)$ for the treatment group and the control group respectively, the difference in RMST between groups can be calculated as

$$\varDelta \mathrm{RMST}={\int}_0^{t^{\ast }}\left[\ {S}_{X_T}(t)-{S}_{X_C}(t)\ \right]\ dt$$

In the simulation study an estimate of the *Δ* RMST was obtained by fitting a RP model under the assumption of PH (RP (PH): time-fixed treatment effects) or allowing for non-PH (RP (TD): time-dependent treatment effects). The *Δ* RMST with *t*^∗^ taken to be the last uncensored observed event time was obtained by predicting the log cumulative hazard functions for the treatment and the control groups over a grid of time values, transforming into the survival functions and integrating over (0, *t*^∗^). Standard errors were estimated using the delta method [29]. By using the last uncensored observed event time, the same events were used for the estimation of *Δ* RMST as were used for the estimates of HR and TR.

#### Time ratio (TR)

The TR is an estimand of treatment effect that arises from direct comparison of the time that elapses until experiencing the outcome event, and for the Weibull data generation model used

$$\mathrm{TR}=\left(\frac{-\ln {\left(S(t)\right)}^{\frac{1}{\gamma }}\exp \left({\beta}_0+{\beta}_1\right)}{-\ln {\left(S(t)\right)}^{\frac{1}{\gamma }}\exp \left({\beta}_0\right)}\right)=\exp \left({\beta}_1\right)$$

In the PH parameterisation of a Weibull regression model, the effect of a covariate is multiplicative by a factor of exp(*β*). In an AFT parameterisation, the effect of a covariate is to accelerate time by a factor of exp(*β*) where the relationship between the coefficients in the two parameterisations is *β*_PH_ =  − *β*_AFT_ × *γ*.

### Methods to assess treatment effect

#### Cox proportional hazards (PH) model

In the Cox PH model the hazard rate for the *i*^*th*^ individual is *h*_*i*_(*t*) = *h*_0_(*t*) exp(*X*_*i*_*β*) with regression coefficients *β* to be estimated and *h*_0_(*t*) denoting the baseline hazard function or event rate [30]. The estimate of treatment effect from the Cox model is obtained by comparing the hazard in the treatment group to the hazard in the control group to obtain the HR. If non-proportional hazards are anticipated, landmark analyses can be obtained by undertaking a Cox analysis conditional on individuals being event free at the pre-specified LM time point *t*_*LM*_. Events prior to *t*_*LM*_ do not contribute to the estimation of the LM HR.

#### Piecewise exponential (PE) regression

The simplest parametric proportional hazards model is the exponential survival model which assumes that the hazard rate is constant over the entire analysis time. To accommodate a non-constant hazard, a useful extension is the piecewise exponential model which allows the time scale to be split into an arbitrary number of intervals each of differing lengths, with a constant hazard rate assumed within each interval. The PE model can be written as *h*_*i*_(*t*) = *λ*_*j*_ exp(*X*_*i*_*β*) where *h*_*i*_(*t*) is the hazard rate for the *i*^*th*^ individual, *λ*_*j*_ is the baseline hazard rate for the *j*^*th*^ follow up interval, *X*_*i*_ is the vector of covariates for the *i*^*th*^ individual and *β* are log hazard-ratios to be estimated. The PE model provides a summary estimate of the HR for the treatment effect for the entire analysis time, or can be extended to provide period-specific estimates of the (*HR*_*j*_) for the treatment effect by including an indicator variable for each period with an interaction with treatment effect.

#### Weibull accelerated failure time (AFT) model

An alternative parameterisation of the Weibull model is the accelerated failure-time model which has the parameterisation ln(*t*_*i*_) = *X*_*i*_*β* + *ϵ*_*i*_ where *ϵ*_*i*_ has an extreme value distribution. Under this parameterisation for the Weibull distribution, the treatment effect is estimated as a summary fixed effect TR in an equivalent manner to the summary HR estimated under the PH assumption.

#### Royston Parmar (RP) models

Royston-Parmar parametric models utilise restricted cubic splines to estimate complex shape functions. The models describe the baseline log cumulative hazard function on the log timescale as a series of cubic spline subfunctions joined at knots with a ‘restriction’ that the first and last subfunctions beyond the boundary knots are linear functions instead of cubic.

The RP PH model can be written as ln(*H*(*t*)) = *s*(ln(*t*)| **γ**_***s***_, **k**_0_) + *X*_*i*_*β* where *s*(ln(*t*)| **γ**_***s***_, **k**_0_) is the restricted cubic spline that is the function of the coefficients of the spline-derived variables (***γ***_***s***_) and the number of knots **k**_**0**_. In the PH context, the RP model is a generalisation of the Weibull distribution where the restricted cubic spline function models the Weibull log cumulative hazard function ln[*H*_0_(*t*)] = ln(*λ*) + *γ* ln(*t*) + *X*_*i*_*β* on the log timescale. The HR and *Δ* RMST for treatment effect can be estimated from this PH model. We assigned 5 degrees of freedom (df) to the baseline distribution which should provide for an adequately flexible fit to a wide variety of survival curves [31]. The *Δ* RMST allowing for TD treatment effects was estimated by including interactions between the treatment variable and additional spline function in the RP model. We assigned 5 df to the baseline distribution as in the PH model, and 2 df to the TD treatment effect to account for possible non-PH.

#### Tests of equal survival functions

Many tests of difference between two survival curves have been proposed that aim to achieve acceptable power under PH and under anticipated non-PH patterns whilst maintaining type I error rates close to the nominal level. Few have become widely accepted as analytical approaches for analysing trials. In this simulation we included tests from two broad categories of test statistics - weighted variants of the LR test designed to improve power under particular non-PH patterns, and omnibus global tests that combine results of several individual tests of significance in an attempt to improve power across a wider range of non-PH patterns. Tests from these two broad categories were identified as the most utilised in recent reviews of analysis methods used in clinical trials with time-to-event outcomes [4, 5].

The classical LR test assesses the null hypothesis that there is no difference between the survival curves of two groups in the probability of an event at any time point over the total survival time period under consideration. The analysis is based on the sum of differences of the estimated hazard function at each observed event time with an implicit equal weighting of one for all event times. Fleming and Harrington proposed a family of weighted tests, the extended FH (*ρ*, *γ*) tests with weighting ${\left[\hat{S}\left(t-\right)\right]}^{\rho }{\left[1-\hat{S}\left(t-\right)\right]}^{\gamma },\rho, \gamma \ge 0$ where $\hat{S}\left(t-\right)$ is the Kaplan-Meier estimate of the survival rate based on the pooled data from the two treatment groups. When *ρ* = 0, *γ* = 0, the FH (0, 0) corresponds to the LR test with equal weights [32]. When *ρ* > *γ*, the test gives more weight to earlier events than to later ones, and when *ρ* < *γ* more weight is given to later events than to earlier ones. In this simulation, the power of the FH tests FH (1, 0), FH (1, 1) and FH (0, 1) weighting early, middle and latter events respectively will be assessed.

The performance of two omnibus tests will be compared in this simulation. The performance of the default form of the versatile test proposed by Karrison [13] considers *Z*_*m*_ = max(| *Z*_1_| , | *Z*_2_| , | *Z*_3_| ) where *Z*_1_, *Z*_2_ and *Z*_3_ are *Z* statistics from the FH (0, 0), FH (1, 0) and FH (0, 1) extended family respectively, and *Z*_*m*_ ∼ N_3_(*μ*, *Σ*) an asymptotic, trivariate normal distribution with **μ** the vector of means and *Σ* the variance-covariance matrix. This combination of *Z* statistics was selected to provide relatively good coverage across the range of likely scenarios encompassing PH, early and late treatment effect scenarios. The second omnibus test which will be assessed in this simulation, the combined test proposed by Royston [12] utilises information from the Cox test and a permutation test based on the maximal squared standardized *Δ* RMST between treatment groups. The motivation for the development of the combined test was to capitalise on the optimal power of the Cox test when the assumption of PH is met, and to provide some insurance should non-PH be present.

### Performance measures

In this simulation study we are interested in assessing the impact of non-constant event rates under two non-PH scenarios on the estimated treatment effect from a range of analysis models. Under PH, the three data-generating models would all result in the same number of events occurring within the specified follow up time. We compared the performance of estimators from an analysis model against the design model knowing that the design model would not necessarily accord with the data-generating model. Discussion of performance measures is in relation to design model using the parameters from the design stage of the trial. This point will be further explained in the context of specific performance measures below.

Power, the first performance measure, was obtained as the proportion of simulations where the *p*-value was less than the nominal significance level *α*. The anticipated power specified at the design stage was 80%. The second performance measure was the scaled treatment effect (STE). The mean treatment effect for each simulation scenario was scaled so that a value of 100% corresponded to the full design-stipulated treatment effect, and a value of 0% would be the anticipated magnitude in the absence of any treatment effect. The scaling was calculated as $\left(1-\mathrm{mean}\left[\hat{HR}\right]\right)/\left(1-H{R}_{design}\right)\times 100$ for the HR estimands, as $\left(\mathrm{mean}\left[\hat{TR}\right]-1\right)/\left(T{R}_{design}-1\right)\times 100$ for the TR estimand, and as $\left(\mathrm{mean}\left[\hat{\varDelta \mathrm{RMST}}\right]\right)/\varDelta {\mathrm{RMST}}_{design}\times 100$ for the *Δ* RMST with the *Δ*RMST_*design*_ value obtained empirically from a large *N* = 250,000 simulation of the design setting. This scaling of treatment effect utilizing the exponentiated measures as reported was designed to allow direct intuitive comparison of the impact of the different simulation scenarios on the magnitude of the three different estimands even though they are a mix of relative and absolute measures, and the beneficial treatment effect can be a value less than 1 (HR) or a value greater than 1 (TR and *Δ*RMST). The final measure, coverage was calculated as the proportion of simulations in which the 100 × (1 − *α*)% confidence interval around analysis model $\hat{\beta}$ included the anticipated *β* from the design model. This allowed assessment of whether the empirical coverage rate approached the desired rate. The anticipated coverage specified at the design stage was 95%.

### Number of simulations

We generated 2000 simulated datasets for each scenario. The Monte Carlo standard errors (MCSEs) for coverage and power are maximized when either 50% power or 50% coverage is observed. In this worst-case scenario, the MCSE for the simulation would be 1.1%. Should coverage and power be optimal at 95 and 80% respectively as implemented under the design scenario, the expected MCSEs would be correspondingly less than 0.5 and 0.9% which we deemed to be acceptable.

## Results

### Type I error

Prior to comparing performance measures such as power for scenarios with a known treatment effect, it is important to assess that analytical approaches are controlling the Type I error level at the same or similar nominal value when there is truly no effect. We compared that empirical Type I errors were maintained reasonably well and similar to other simulation studies [33, 34]. Additional detail of the Type I error assessment is presented in Additional file 1.

### Lag until treatment effect

#### Power of regression model approaches

**Fig. 3:**
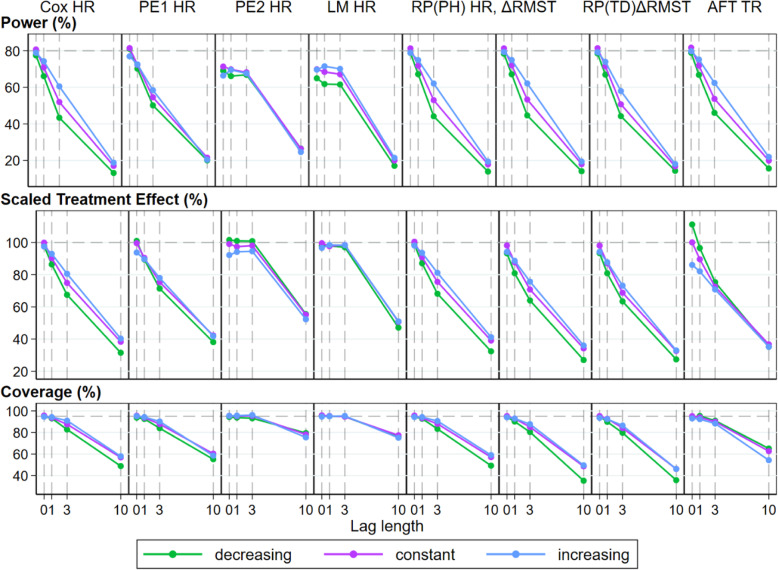
Performance measures of regression-based approaches for treatment effect estimation under increasing lag until effect DGM. The power (%), scaled treatment effect magnitude (%) and coverage (%) are presented as relative to that anticipated at the design stage of the trial assuming PH. Lag period lengths investigated were *t*_*lag*_ = 0, 1, 3 and 10 months within the maximum follow-up time *t* = 50, with the setting *t*_*lag*_ = 0 representing PH

Figure 3 presents the simulation results investigating the effect of lag times for eight different modelling approaches to estimating the HR, *Δ* RMST and TR. For an indication of data maturity, the average number of events for the constant event rate during the no effect period was 10, 26 and 65% of the total number of events observed for the lag times of 1, 3 and 10 months respectively. For the decreasing hazard event rate, the average number of events during the no-effect period were 14, 34 and 71%, and for the increasing hazard event rate, the average number of events during the no-effect period were 7, 21 and 60% of the total number of events observed for the lag times of 1, 3 and 10 months respectively. A summary of event numbers during the inactive and active phases of treatment effect under this non-PH scenario is presented in Supplementary Table S2 in Additional file 1.

In the top panel of Fig. 3 for the first scenario with no lag to effect (*t*_*lag*_ = 0, the PH scenario), we observed power very close to the design model value of 80% for all estimates of treatment effect. There was lower power for the two period-specific power estimates (PE2 and LM) resulting from the smaller number of events used in the estimation of HR after the prespecified cut points of *t*_*PE*_ and *t*_*LM*_ were applied. For all methods, there was an appreciable loss of power in these non-PH scenarios. This loss of power was present even when *t*_*lag*_ = 1 with greater loss of power observed with increasing lag times.

The impact of non-constant event rates in the presence of non-PH can also be clearly observed, with the difference in power most differentiated when *t*_*lag*_ = 3.In general, an increasing event rate slightly attenuated the loss of power as a result of fewer events occurring during the lag period, relative to the number of events observed under a constant event rate. Conversely, the losses in power observed under a decreasing event rate in the presence of a lag until effect were magnified as a result of more events occurring during the period where the treatment had no effect. This pattern of relative power loss with non-constant event rates was observed for the HR, TR and *Δ* RMST.

#### Scaled treatment effects (STE) estimates of regression model approaches

The middle panel of Fig. 3 presents the STE results. In the scenario of no lag until treatment effect (*t*_*lag*_ = 0) estimates close to the design model values are observed except for the *HR* from the PE2 model and the *TR* from the AFT model. For these two estimators, an increasing event rate resulted in a lower STE under PH whilst a decreasing event rate resulted in a higher STE. The presence of any lag period resulted in STE of decreased average magnitude as there were less events occurring during the period where the treatment was effective. Compared to a constant event rate, an increasing event rate was able to partially ameliorate this decrease in STE whilst a decreasing event rate compounded the decrease.

#### Coverage of regression model approaches

In the bottom panel of Fig. 3, coverage of the estimators for the treatment effect used in the design model is presented. Under PH, we observed coverage at, or very close to, the design model value of 95%. In the presence of a lag until treatment effect, there was a consistent decrease in the observed coverage with increasing lag for all methods. The presence of non-constant event rates has less impact on this performance measure. The summary estimates for bias, coverage and power with the Monte Carlo standard errors (MCSEs) for simulations in the presence of a lag until treatment for the decreasing, constant and increasing baseline hazards are presented in Supplementary Tables S3, S4 and S5 respectively in Additional file 1.

#### Power of the tests of equal survival curves

**Fig. 4:**
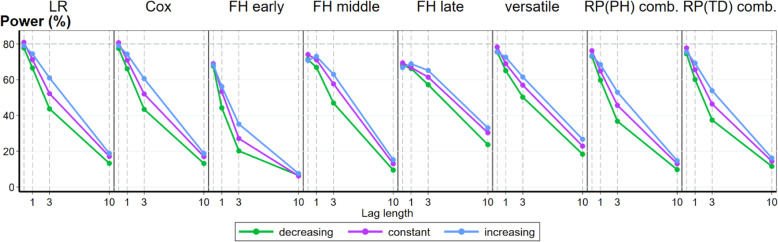
Power of tests of equal survival function under increasing lag until effect DGM. Effect of non-constant event rates on the power of seven tests of equal survival function. The power of the *z*-test for the HR treatment effect from the Cox PH model is included in the panel as a comparator. Lag period lengths investigated were *t*_*lag*_ = 0, 1, 3 and 10 months within the maximum follow-up time *t* = 50, with the setting *t*_*lag*_ = 0 representing PH

Figure 4 presents the results for seven tests of equal survival functions compared in the simulation. The power of the *z*-test for the treatment effect from the Cox model is included in the panel as a comparator. Results are broadly similar to that observed for the modelling approaches. In the scenario equivalent to PH, the LR, Cox, versatile and combination tests achieved power values close to the design model value of 80%. The power dropped swiftly with increasing lag times. The decreased or increased loss of power observed could be substantial for some tests exceeding ±10% of the power observed under a constant event rate depending on the length of the lag effect under consideration.

### Early effect that ceases

The early effect that ceases non-PH scenario is the inverse in treatment effect timing to the lag until treatment effect. The performance measures for the early effect that ceases non-PH scenario were similarly the converse to that observed in the lag until treatment effect non-PH simulations. In summary, increasing losses of power and decreased magnitude of the treatment effects and coverage were observed as the length of the treatment effect period decreased. Relative to a constant event rate, more events occurred during the early effective period under a decreasing baseline hazard resulting in some offset of the losses in performance measures observed. Under an increasing event rate, some reduction of the losses observed under the constant event rate were observed. This pattern of relative loss was observed for all three estimands and similar losses in power were observed in the tests of equal survival curves as were observed for the regression-based approaches. Results are described in more detail in the Supplementary Results section in Additional file 1.

## Discussion

We have shown that when time-dependent treatment effects are anticipated, then non-PH and non-constant event rates should both be considered at the time of designing a trial. The adverse impact of non-PH on power can be further exacerbated or potentially ameliorated by the shape of the baseline hazard. Non-proportionality of treatment effects has been increasingly observed in clinical trials [16, 35]. New treatments being assessed are often more complex, involving comparison of new oncology treatments with different biological time courses of action, or comparing treatments with different mechanisms of action such as surgical versus chemotherapeutic approaches, or involving the use of composite outcomes - multiple endpoints jointly assessed as a primary outcome - all increasing the chance of encountering non-PH [36]. Due to increased oversight and increased awareness of the importance of personalised medicine, trials are often longer in planned follow up, with larger numbers of participants included to allow for greater assessment of differently responsive sub-populations within them. Trials of longer duration allow a greater opportunity for non-PH to arise over time, and larger numbers of events enable assessment of the presence of any non-PH to be more conclusive. The potential impact of non-PH has been brought into focus due to these longer, larger trials being conducted [33, 37, 38]. For these trials, non-constant event rates will also be more likely to be observed, yet the interplay between non-PH and the shape of the baseline hazard rates has received little attention before now, despite the reasonable anticipation that it could also to have important design implications for clinical trials.

### Comparison of power of tests of survival curve difference

In our results, when there was a lag until treatment effect, the best performing test of survival curve difference in terms of maintaining power under PH and shorter and longer lengths of effective treatment time was the versatile test. The FH late test was more powerful when there was longer lags until effect, but was less powerful under shorter lags and PH scenarios more likely to be encountered in trials compared to the versatile test. When there is an early effect that ceases, the versatile test closely followed by the RP (TD) combined test would be the recommended option. Increasing and decreasing event rates affected the power of the tests compared to a constant event rate, in accordance with the timing of when events were likely to be observed with respect to the periods of effective treatment. Power was increased when relatively more events occurred during effective treatment times and decreased when relatively fewer events occurred during effective treatment times. At the time of designing a trial, if assumptions about the presence and form of non-PH are not made, then our results suggest that the versatile test covering PH, early and late forms of non-PH is recommended as a pre-specified analysis method. This test will retain power under more modest levels of non-PH whilst maintaining near nominal power under PH and will be less adversely affected by non-constant event rates.

Our results accord with similar comparative studies published recently that focus on tests of survival curve difference [33, 34, 38]. As part of Cross-Pharma Non-Proportional Hazards (NPH) working group, Lin et al. (2020) compared nine tests of survival curve difference in the presence of non-PH covering the LR and weighted LR tests, weighted Kaplan-Meier based tests (incorporating the RMST) and combination tests [38]. Royston and Parmar also included a similar range of tests covering weighted LR tests and composite tests based on their own [39] and Karrison’s work [13]. Jimenez et al. (2019) investigated the properties of the weighted LR tests in the presence of trials with delayed effects [34]. There is substantial overlap between the tests included in this simulation study and the three other studies, with similar focus on early (treatment effects that cease) and late (lag until treatment effect) forms of non-PH. For the tests of survival curve difference in the presence of any non-PH, broadly similar conclusions were reached by all four studies: that what might have been regarded as minimal amounts of non-PH - whether expressed in terms of information fraction or percent of study duration - can noticeably affect the power to detect survival curve differences, and for the trials assessing different forms of non-PH, there is no consistently powerful test across all non-PH scenarios. Forms of a versatile test combining information from multiple weighted LRs were the recommended form of pre-specified test when considering early and late non-PH scenarios [33, 38]. When late non-PH is the only consideration, LR tests weighted to emphasize late differences are recommended to maintain higher power albeit at the expense of slight Type I error rate inflation [34].

### Treatment effect estimands - HR v RMST v AFT

We compared three different estimands for treatment effect - the HR, the TR and *Δ* RMST. There have been many studies comparing these estimands and variants of them for their use in research with TTE outcomes [14, 16, 20, 21, 40–43]. There are strengths and limitations in their usage - relative measures such as the HR and TR do not contain any information about the absolute effect and can be challenging to interpret and communicate the survival benefit observed. Estimates provided in a time-based metric such as the TR and the RMST expressed either as a ratio or a difference, can be considered more interpretable for a wider audience. The *Δ* RMST has an additional advantage of being a summary measure of survival time distribution that does not rely on the PH assumption although it does require specification of the cutoff timepoint. In this work, we estimated *Δ* RMST using both the last uncensored event occurrence as the cutoff time following recommended practice [25] as well as the maximum follow up time (*t* = 50). By design, the last uncensored event would have been expected to occur at a time very close to the maximum follow up time. As a consequence of these design choices, we observed essentially no differences within simulation error in any of the performance measures of *Δ* RMST using either the last uncensored event cut off or the maximum follow up time, and hence presented the results for the last uncensored event time cutoff only in the interests of clarity.

For this work, the three estimands we compared were broadly similar across the non-PH scenarios in terms of the power, magnitude of treatment effect estimate and coverage values benchmarked to the values specified by the design model. Judicious selection of designated cutpoints for no effect (PE2) or landmark timepoints (LM) could result in improved estimates of treatment effect magnitude using the period-specific analysis methods in the presence of a lag until effect non-PH, but also resulted in decreased power if there was PH. Similarly, the *Δ* RMST could be assessed at a number of prespecifed clinically relevant time points in order to provide insight into how treatment effects may change with follow up time. The potential for increased Type I error that may arise from multiple comparisons would need to be monitored, and empirical measures to correct for any inflation would have to be incorporated into the trial design [34].

The impact of non-constant event rates in the presence of non-PH was to partially diminish or further exacerbate losses in power and treatment effect magnitude. When time-dependent treatment effects are present, there is no single summary measure that can adequately describe the treatment benefit. Analysis methods such as the RP models which allow for the shape of the baseline hazard make it possible to more fully explore the timing and magnitude of any treatment effect either graphically or in a series of time period-based estimates.

### Designing trials with non-constant event rates in the presence of non-PH

Simulation studies can only ever include a limited range of scenarios. It is critical that selections are made so as to provide insight on the wider and varied spectrum of scenarios involving non-PH and non-constant event rates that are likely to be encountered in real RCTs. We restricted attention to simplified forms of non-PH - piecewise constant HRs with a single change point - comparing PH with early and late forms of non-PH. Change points were placed at times that enabled us to observe effects over a large proportion of calculated power values with magnitudes of treatment effect ranging from the design-stipulated to nearly null estimates. Hence our results may not generalize to more complex forms of non-PH. When choosing non-constant event rates, we aimed to cover clinically plausible values of the shape parameter in our data-generating Weibull model that are modest and hence might be assumed to be ‘close enough’ to constant at the design stage of a trial. More extreme settings could have been chosen and the impacts on power and effect estimation would have been exaggerated to the point of being quite drastic; however, we felt that this would represent uncommon scenarios in practice. Our simulations also featured almost complete follow up of all events before undertaking analysis which, whilst unrealistic in some applications, resulted in almost identical numbers of total events being observed in each scenario, and hence provided a fair basis for comparison. We did not cover the effects of censoring and enrolment rates, nor did we investigate the effect of adjusting sample size and follow up times all of which impact on the interplay of non-PH and event rates and may need to be considered in practice. Sample size calculation options are available for specific forms of non-PH [44], parametric event rates [45, 46], piecewise models that allow for different treatment effects within multiple ‘stages’ of a planned trial [47, 48]. However, the most flexible approach to take is to base the sample size on simulation [49, 50]. These approaches have been employed in multi-arm multi-stage and other forms of adaptive trial design. The additional complexity includes the need for prior specification of additional parameters and a higher degree of programming skill to explore scenarios covering anticipated event rates and the direction and timing of non-proportionality.

## Conclusions

The mechanisms of action of treatments on time to event outcomes may require nuanced definitions of treatment effectiveness that go beyond simple single summary estimates assuming proportional hazards. Our simulations found that even small deviations from proportionality can result in substantial observed loss of power using standard analysis methods that are maximally powerful under a PH assumption, and this loss can be exacerbated in the presence of non-constant event rates. It is a desirable strategy to design trials to use analysis methods that can accommodate delayed treatment effects, or early treatment effects that cease if these are to be anticipated with the treatment under study. This however requires decisions on what test to employ and what estimand(s) will be the target. Our simulations provide some guidance on this choice. In practice, new trials may require the use of bespoke simulation studies to guarantee that power is maintained under a range of plausible scenarios consistent with expected mechanisms of treatment action and allowing for departures from non-constant underlying event rates.

## Supplementary Information

**Additional file 1.**

**Additional file 2.**

## Data Availability

All data generated or analysed during this study are included in this published article [Additional_file_2.pdf].

## Abbreviations

RCT: Randomised controlled trial
PH: Proportional hazards
LR: Logrank
HR: Hazard ratio
FH: Fleming-Harrington
RMST: Restricted mean survival time
AFT: Accelerated failure time
RP: Royston -Parmar
LM: Landmark
TR: Time ratio
DGM: Data-generating model
PE: Piecewise exponential
TD: Time-dependent
STE: Scaled treatment effect
MCSE: Monte Carlo standard error
