# Supplementary material for: Impact of a non-constant baseline hazard on detection of time-dependent treatment effects: a simulation study
Source: BMC Med Res Methodol. 2021 Aug 28;21:177. doi: 10.1186/s12874-021-01372-0 (PMC8399795; doi:10.1186/s12874-021-01372-0)
Supplement: Supplementary file 1 — Additional file 1. [file 12874_2021_1372_MOESM1_ESM.pdf]

For: Impact of a non-constant baseline hazard on detection of time-dependent treatment effects

Supplementary Information

Supplementary Methods

Data-generating processess for simulation scenarios

Early effect that ceases

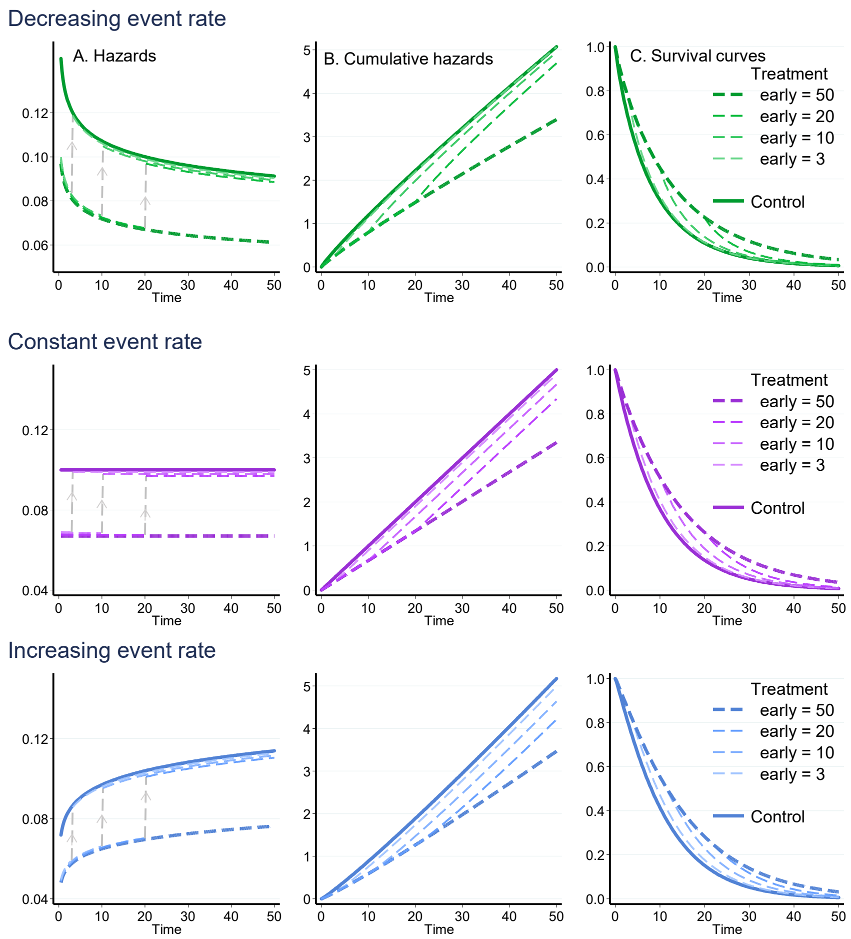

Supplementary Figure S1: Baseline hazard function, cumulative hazard curves and survival curves for the early effect that ceases non-PH under three event rates. The control group is indicated by the lightest dashed line and the treatment group is shown by the darkest solid lines. Increasing effect times of 3, 10 and 20 months are indicated by the increased shading and decreased dashed lines. Decreasing, constant and increasing event rate scenarios are indicated by the green, purple and blue lines respectively.

Supplementary Results

Type I error

When comparing performance measures such as power for the stipulated PH and non-PH scenarios with a known treatment effect, it is important to assess all analytical approaches are controlling the Type I error level at the same or similar nominal value when there is truly no effect. We compared the empirical type I error of the tests of regression-based treatment effect estimate and equal survival function under the null treatment effect by simulation. In these simulations, there was no treatment effect (ie HR =1) in both periods specified by the data- generating models. Pooled replicates for each event rate type and all change point times are presented in Table S1. For the majority of the tests, the empirical Type I errors are within or close to the nominal two-sided 5% significance level. The Type I error of the RP(PH) combined test is conservative under both types of non-PH. A minor Type I error inflation is observed for the FH(0,1) test weighted for late effects (Type I error: 5.7% (95% CI 5.5%, 5.8%)), with even smaller increases in the Type I error above the nominal level also being observed for the versatile test and the regression coefficient estimates for the RP(PH) ΔRMST, the RP(TD)ΔRMST and the AFT TR. Similar minor increases in Type I error rate have been reported in other simulation studies comparing the power of tests for treatment effect under non-PH scenarios.

Supplementary Figures S2 and S3 further present the results of this empirical assessment of Type I error by the decreasing, constant and increasing event rate scenarios using the change points from the lag to effect and early effect that ceases non-PH scenario under investigation for each of the analysis methods. Comparisons of the power of the analysis methods presented below needs to be undertaken with the conservative Type I error for the RP(PH) combined test, and minor inflation of the empirical Type I errors for versatile test and the regression coefficient estimates for the RP(PH) ΔRMST, the RP(TD) ΔRMST and the AFT TR in mind.

Table S1: Empirical Type I error (%) of the test of treatment effect or equal survival functions

		Size (95% CI)
Estimands of treatment effect		
Cox Proportional Hazards HR	Cox HR	5.1 (5.0, 5.3)
Piecewise Exponential HR	PE1 HR	5.2 (5.0, 5.3)
Royston-Parmar (PH) HR	RP(PH)	5.3 (5.1, 5.5)
Royston-Parmar (PH) ΔRMST	RP(PH) ΔRMST	5.4 (5.2, 5.6)
Royston-Parmar (TD) ΔRMST	RP(TD) ΔRMST	5.4 (5.3, 5.6)
Accelerated Failure Time model TR	AFT TR	5.4 (5.2, 5.5)
Tests of equal survival functions		
Logrank	LR	5.1 (5.0, 5.3)
Fleming Harrington (1,0) early effects	FH early	5.1 (4.9, 5.2)
Fleming Harrington (1,1) middle effects	FH middle	5.1 (4.9, 5.2)
Fleming Harrington (0,1) late effects	FH late	5.7 (5.5, 5.8)
Versatile test	versatile	5.4 (5.3, 5.6)

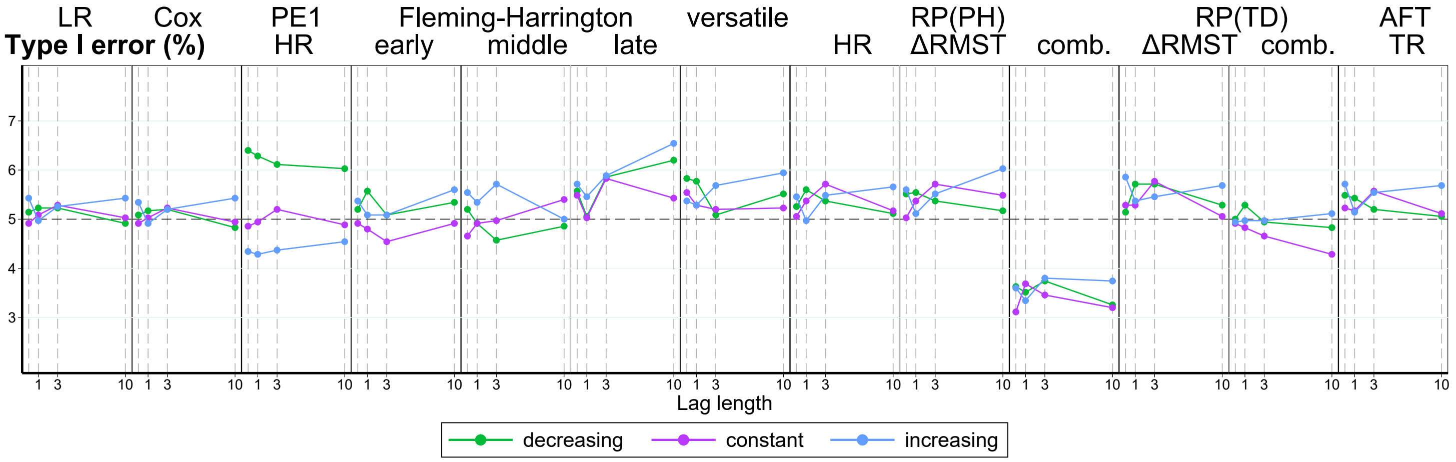

Supplementary Figure S2: Empirical type I error of the tests of regression-based treatment effect estimate and equal survival functions under the null treatment effect by event rate for change point times used in the increasing lag until effect data-generating model. Decreasing, constant and increasing event rate scenarios are indicated by the green, purple and blue lines respectively.

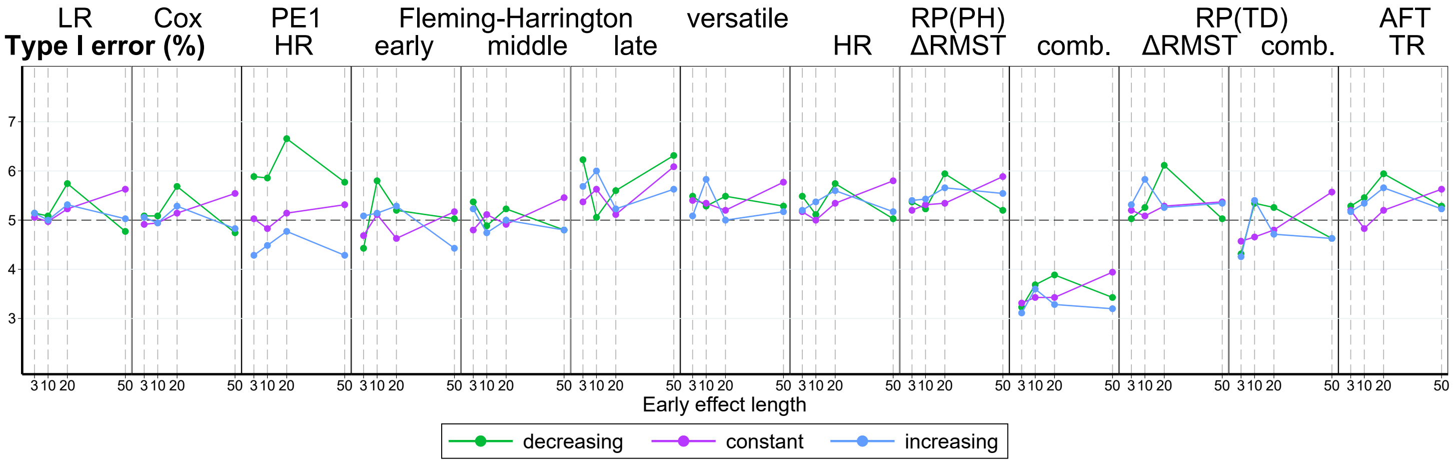

Supplementary Figure S3: Empirical type I error of the tests of regression-based treatment effect estimate and equal survival functions under the null treatment effect by event rate for change point times used in the early effect that ceases data-generating model. Decreasing, constant and increasing event rate scenarios are indicated by the green, purple and blue lines respectively.

Lag until treatment effect

Table S2: Summary of event numbers during the inactive and active phases of the treatment effect in the simulation investigating the effect of a lag until treatment effect

Lag until effect	Event rate	Number events inactive phase Mean (range)	Number events active phase Mean (range)	Total number of events (N)
	Decreasing	N/A	198 (190,202)	198 (190,202)
None	Constant	N/A	198 (188,202)	198 (188,202)
	Increasing	N/A	198 (189,202)	198 (189,202)
	Decreasing	28 (11,46)	170 (151,188)	198 (191,202)
One	Constant	19 (6,35)	179 (188,202)	198 (190,202)
	Increasing	14 (2,25)	185 (170,198)	198 (190,202)
	Decreasing	67 (46,95)	131 (103,153)	198 (190,202)
Three	Constant	52 (34,72)	146 (128,167)	198 (189,202)
	Increasing	42 (26,69)	156 (131,172)	198 (190,202)
	Decreasing	141 (118,161)	58 (38, 79)	199 (192,202)
Ten	Constant	128 (107,150)	71 (49, 93)	199 (192,202)
	Increasing	118 (98,143)	81 (56,100)	199 (192,202)

Table S3: Bias (MCSE), % Coverage (MCSE) and % Power (MCSE) for the scenario investigating the lag until treatment effect that ceases for the decreasing baseline hazard

		Hazard Ratio					ΔRMST		Time Ratio
	Effect time	Cox PH	PE1	PE2	LM	RP(PH)	RP(PH)	RP(TD)	Weibull AFT
Bias	Zero	0.01 (0.003)	-0.01 (0.003)	-0.02 (0.004)	0.00 (0.004)	0.00 (0.003)	-0.3 (0.03)	-0.3 (0.03)	0.04 (0.004)
	One	0.06 (0.003)	0.05 (0.003)	-0.01 (0.004)	0.00 (0.004)	0.06 (0.003)	-0.9 (0.03)	-0.9 (0.03)	-0.01 (0.004)
	Three	0.14 (0.003)	0.13 (0.003)	-0.02 (0.004)	0.00 (0.004)	0.14 (0.003)	-1.6 (0.04)	-1.7 (0.04)	-0.09 (0.004)
	Ten	0.29 (0.003)	0.26 (0.004)	0.19 (0.004)	0.22 (0.004)	0.28 (0.003)	-3.3 (0.03)	-3.3 (0.03)	-0.25 (0.004)
Coverage	Zero	94.8 (0.5)	93.8 (0.5)	94.3 (0.5)	95.4 (0.5)	94.6 (0.5)	94.3 (0.5)	94.8 (0.5)	93.8 (0.5)
	One	92.8 (0.6)	92.4 (0.6)	93.9 (0.5)	94.9 (0.5)	92.6 (0.6)	90.2 (0.7)	89.9 (0.7)	95.3 (0.5)
	Three	82.4 (0.9)	83.6 (0.8)	93.4 (0.6)	94.7 (0.5)	82.9 (0.8)	80.4 (0.9)	79.7 (0.9)	90.9 (0.6)
	Ten	48.0 (1.1)	54.4 (1.1)	79.1 (0.9)	75.5 (1.0)	48.7 (1.1)	35.3 (1.1)	35.8 (1.1)	65.2 (1.1)
Power	Zero	77.5 (0.9)	81.1 (0.9)	69.2 (1.0)	64.9 (1.1)	78.8 (0.9)	78.3 (0.9)	78.5 (0.9)	78.9 (0.9)
	One	66.1 (1.1)	70.2 (1.0)	66.2 (1.1)	61.8 (1.1)	67.2 (1.0)	67.2 (1.1)	66.9 (1.1)	66.8 (1.1)
	Three	43.4 (1.1)	50.2 (1.1)	66.8 (1.1)	61.5 (1.1)	44.2 (1.1)	44.7 (1.1)	44.3 (1.1)	46.0 (1.1)
	Ten	13.1 (0.8)	20.0 (0.9)	26.5 (1.0)	17.0 (0.8)	13.9 (0.8)	14.1 (0.8)	14.4 (0.8)	15.6 (0.8)

Table S4: Bias (MCSE), % Coverage (MCSE) and % Power (MCSE) for the scenario investigating the lag until treatment effect that ceases for the constant baseline hazard

		Hazard Ratio					ΔRMST		Time Ratio
	Effect time	Cox PH	PE1	PE2	LM	RP(PH)	RP(PH)	RP(TD)	Weibull AFT
Bias	Zero	0.00 (0.003)	0.00 (0.003)	0.00 (0.004)	0.00 (0.004)	-0.01 (0.003)	-0.1 (0.03)	-0.1 (0.03)	0.00 (0.003)
	One	0.04 (0.003)	0.04 (0.003)	0.01 (0.004)	0.00 (0.004)	0.04 (0.003)	-0.6 (0.04)	-0.6 (0.04)	-0.04 (0.003)
	Three	0.11 (0.003)	0.11 (0.003)	0.00 (0.004)	0.00 (0.004)	0.11 (0.003)	-1.3 (0.04)	-1.4 (0.04)	-0.10 (0.003)
	Ten	0.26 (0.003)	0.25 (0.003)	0.19 (0.004)	0.21 (0.004)	0.26 (0.003)	-3.0 (0.03)	-3.1 (0.03)	-0.24 (0.003)
Coverage	Zero	95.7 (0.5)	95.7 (0.5)	95.4 (0.5)	95.8 (0.4)	95.7 (0.5)	95.2 (0.5)	95.2 (0.5)	95.2 (0.5)
	One	93.4 (0.6)	93.7 (0.5)	95.2 (0.5)	95.3 (0.5)	93.2 (0.6)	92.9 (0.6)	92.8 (0.6)	94.1 (0.5)
	Three	87.3 (0.7)	87.5 (0.7)	95.3 (0.5)	94.8 (0.5)	87.4 (0.7)	85.1 (0.8)	84.2 (0.8)	90.0 (0.7)
	Ten	56.3 (1.1)	59.4 (1.1)	77.5 (0.9)	76.7 (0.9)	56.5 (1.1)	48.6 (1.1)	46.3 (1.1)	62.7 (1.1)
Power	Zero	80.7 (0.9)	81.4 (0.9)	71.3 (1.0)	69.8 (1.0)	81.3 (0.9)	81.3 (0.9)	81.4 (0.9)	81.7 (0.9)
	One	70.9 (1.0)	72.3 (1.0)	69.8 (1.0)	68.4 (1.0)	71.8 (1.0)	72.1 (1.0)	71.6 (1.0)	71.8 (1.0)
	Three	52.0 (1.1)	54.5 (1.1)	68.2 (1.0)	67.2 (1.1)	53.0 (1.1)	53.3 (1.1)	50.8 (1.1)	53.7 (1.1)
	Ten	17.0 (0.8)	21.5 (0.9)	26.4 (1.0)	20.0 (0.9)	17.9 (0.9)	18.0 (0.9)	16.8 (0.8)	20.0 (0.9)

Table S5: Bias (MCSE), % Coverage (MCSE) and % Power (MCSE) for the scenario investigating the lag until treatment effect that ceases for the increasing baseline hazard

		Hazard Ratio					ΔRMST		Time Ratio
	Effect time	Cox PH	PE1	PE2	LM	RP(PH)	RP(PH)	RP(TD)	Weibull AFT
Bias	Zero	0.01 (0.003)	0.03 (0.003)	0.03 (0.003)	0.01 (0.004)	0.01 (0.003)	-0.3 (0.04)	-0.3 (0.04)	-0.05 (0.003)
	One	0.03 (0.003)	0.05 (0.003)	0.02 (0.003)	0.00 (0.004)	0.03 (0.003)	-0.5 (0.03)	-0.6 (0.03)	-0.06 (0.003)
	Three	0.09 (0.003)	0.10 (0.003)	0.02 (0.003)	0.00 (0.004)	0.08 (0.003)	-1.1 (0.03)	-1.2 (0.04)	-0.10 (0.003)
	Ten	0.25 (0.003)	0.25 (0.003)	0.20 (0.004)	0.21 (0.004)	0.25 (0.003)	-2.9 (0.03)	-3.0 (0.03)	-0.25 (0.003)
Coverage	Zero	94.4 (0.5)	95.1 (0.5)	95.5 (0.5)	94.8 (0.5)	94.3 (0.5)	94.1 (0.5)	93.6 (0.5)	93.1 (0.6)
	One	94.1 (0.5)	94.3 (0.5)	95.4 (0.5)	95.1 (0.5)	94.1 (0.5)	92.9 (0.6)	92.4 (0.6)	92.3 (0.6)
	Three	90.7 (0.7)	89.8 (0.7)	96.1 (0.4)	95.3 (0.5)	90.3 (0.7)	87.8 (0.7)	86.4 (0.8)	88.3 (0.7)
	Ten	57.1 (1.1)	57.8 (1.1)	74.7 (1.0)	74.7 (1.0)	58.3 (1.1)	49.5 (1.1)	46.2 (1.1)	54.3 (1.1)
Power	Zero	78.9 (0.9)	76.9 (0.9)	66.4 (1.1)	69.7 (1.0)	78.9 (0.9)	79.3 (0.9)	79.2 (0.9)	79.8 (0.9)
	One	74.3 (1.0)	72.6 (1.0)	69.5 (1.0)	71.5 (1.0)	74.9 (1.0)	74.9 (1.0)	73.9 (1.0)	75.2 (1.0)
	Three	60.5 (1.1)	58.5 (1.1)	67.6 (1.0)	70.1 (1.0)	62.1 (1.1)	62.2 (1.1)	58.0 (1.1)	62.5 (1.1)
	Ten	18.8 (0.9)	20.4 (0.9)	24.6 (1.0)	21.5 (0.9)	19.4 (0.9)	19.4 (0.9)	18.0 (0.9)	22.0 (0.9)

Supplementary Results cont'd

Early effect that ceases

Power of the regression model approaches

Figure S4 presents the results for the non-PH scenario of an early effect that ceases. Seven different modelling approaches were compared. For the constant hazard event rate scenario, the average number of events during the effective treatment period were 22%, 56% and 82% of the total number of events observed for the early effect times of three, ten and twenty months respectively. For the decreasing hazard event rate, the average number of events during the period when there was an early effect were 28%, 63% and 85%, and for the increasing hazard event rate, the average number of events during the effective period were 18%, 52% and 80% of the total number of events observed for the early effect times of three, ten and twenty units respectively. Supplementary Table S6 presents a summary of event numbers during the active and inactive phases of treatment effect for this early effect that ceases non-PH scenario.

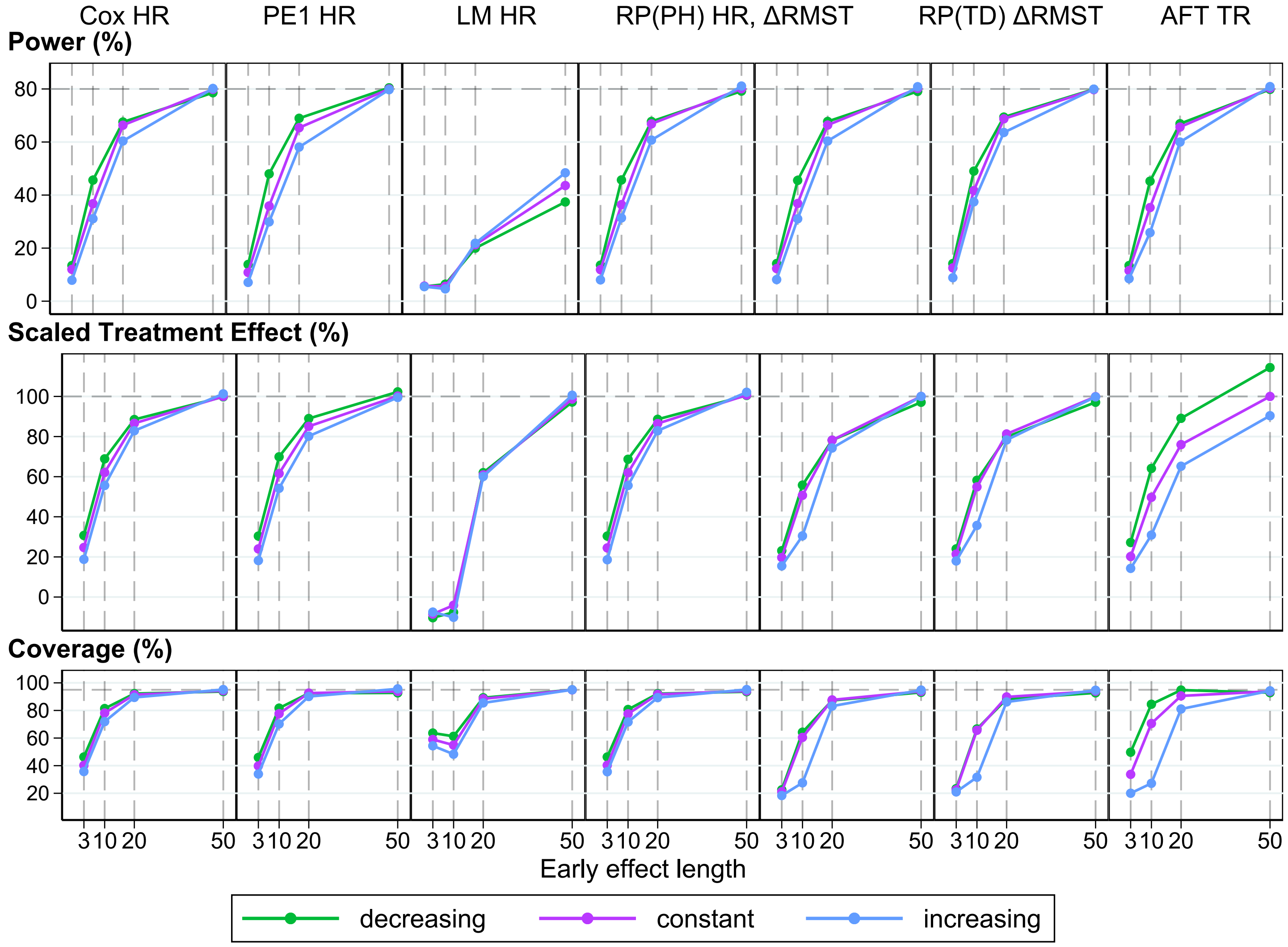

Supplementary Figure S4: The power (%), scaled treatment effect magnitude (%) and coverage (%) are presented as relative to that anticipated at the design stafe of the trial assuming PH. The early effect period lengths investigated were $t_{early} = 3, 10, 20$ and 50 months, with the setting $t_{early} = 50$ representing PH.

When the treatment was constantly effective throughout the follow up period ($t_{early} = 50$) equivalent to a PH data generating model, we observed power at or very close to the design model values of 80% for all estimates of treatment effect except for the LM method. There was substantial decreased power for this period-specific estimate partly due to less than half of the events

Table S6: Summary of event numbers during the active and inactive phases of the treatment effect in the simulation investigating an early treatment effect that ceases

Early effect time	Event rate	Number events active phase Mean (range)	Number events inactive phase Mean (range)	Total number of events (N)
	Decreasing	57 (38,78)	143 (121,163)	201 (195,202)
Three	Constant	45 (28,66)	156 (135,173)	201 (196,202)
	Increasing	36 (15,54)	165 (146,184)	201 (194,202)
	Decreasing	126 (103,148)	75 (53, 95)	198 (191,202)
Ten	Constant	113 (90,138)	87 (64,110)	198 (190,202)
	Increasing	104 (77,128)	97 (73,125)	198 (190,202)
	Decreasing	168 (147,184)	32 (16,54)	198 (190,202)
Twenty	Constant	162 (142,181)	38 (19,57)	198 (189,202)
	Increasing	158 (140,175)	42 (25,61)	198 (190,202)
	Decreasing	198 (188,202)	N/A	198 (188,202)
Fifty	Constant	198 (191,202)	N/A	198 (191,202)
	Increasing	198 (191,202)	N/A	198 (192,202)

being used in the estimation of HR after the prespecified cutpoint of $t_{LM} = 10$ was applied under all event rates, and partly due to the inclusion of more events from the no treatment effect period. For all methods, there was an appreciable loss of power in the early effect non-PH scenario. A decreasing event rate was able to offset the lower power seen as a result of fewer events occurring during the period when the treatment effect had ceased, relative to the number of events observed under a constant event rate. Conversely, the losses in power observed under an increasing event rate in the presence of an early effect that ceases were greater as a result of more events occurring during the period where the treatment had no effect. This pattern of relative power loss was observed for all three estimands.

Scaled Treatment Effect (STE) estimates of regression model approaches

The results comparing the magnitude of treatment effect estimates are presented in the middle panel of Figure S4. For the STE under the PH scenario ($t_{early} = 50$), estimates close to the design model values are obtained for the HR and ΔRMST estimands. Non-constant event rates affect the magnitude of the TR estimated from an AFT model. A decreasing event rate resulted in STEs greater than were observed with a constant event rate, and an increasing event rate resulted in STEs lower than estimated under constant event rates.

Coverage of regression model approaches

Coverage of the estimators for the treatment effect used in the design model is presented in the bottom panel of Figure S4. Under PH, coverage at the design model value of 95% was observed when the treatment effect persisted throughout the analysis period ($t_{early} = 50$). The presence of an early effect that ceases quickly causes a dramatic decrease in the observed coverage. In contrast, having a treatment that stops being effective later has far less impact and most of the nominal coverage is maintained. Non-constant event rates have minimal impact on coverage for the estimates of HR, but for an increasing event rate, estimates of ΔRMST were more affected. The effect of non-constant event rates was most noticeable for the coverage estimates for the TR from an AFT model, consistent with the observed effect of non-constant event rates on the STEs. The summary estimates for bias, coverage and power with the Monte Carlo standard errors (MCSEs) for simulations under this scenario for the decreasing, constant and increasing baseline hazards are presented in Supplementary Tables S7, S8 and S9 respectively.

Table S7: Bias (MCSE), % Coverage (MCSE) and % Power (MCSE) for the scenario investigating the early treatment effect that ceases for the decreasing baseline hazard

		Hazard Ratio					ΔRMST		Time Ratio
	Effect time	Cox PH	PE1	PE2	LM	RP(PH)	RP(PH)	RP(TD)	Weibull AFT
Bias	Three	0.29 (0.003)	0.29 (0.003)	0.41 (0.006)	0.40 (0.006)	0.29 (0.003)	-3.5 (0.03)	-3.5 (0.03)	-0.29 (0.003)
	Ten	0.14 (0.003)	0.14 (0.003)	0.40 (0.006)	0.40 (0.006)	0.14 (0.003)	-2.1 (0.03)	-2.0 (0.03)	-0.14 (0.003)
	Twenty	0.06 (0.003)	0.06 (0.003)	0.16 (0.005)	0.15 (0.006)	0.06 (0.003)	-1.1 (0.03)	-1.1 (0.03)	-0.05 (0.003)
	Fifty	0.01 (0.003)	-0.01 (0.004)	-0.01 (0.006)	0.00 (0.006)	0.00 (0.003)	-0.3 (0.04)	-0.3 (0.04)	0.04 (0.004)
Coverage	Three	45.7 (1.1)	45 (1.1)	62.5 (1.1)	63.5 (1.1)	45.3 (1.1)	22.5 (0.9)	23.4 (0.9)	49.8 (1.1)
	Ten	80.8 (0.9)	81 (0.9)	60.4 (1.1)	60.7 (1.1)	80.1 (0.9)	64.3 (1.1)	66.6 (1.1)	84.5 (0.8)
	Twenty	92.1 (0.6)	92.3 (0.6)	88.9 (0.7)	89.1 (0.7)	91.8 (0.6)	87.3 (0.7)	88.3 (0.7)	94.8 (0.5)
	Fifty	93.7 (0.5)	92.7 (0.6)	94.5 (0.5)	95 (0.5)	93.6 (0.5)	93.1 (0.6)	92.8 (0.6)	92.9 (0.6)
Power	Three	13.4 (0.8)	13.9 (0.8)	6.1 (0.5)	5.6 (0.5)	13.6 (0.8)	14.2 (0.8)	14.2 (0.8)	13.4 (0.8)
	Ten	45.7 (1.1)	48.0 (1.1)	6.6 (0.6)	6.4 (0.5)	45.7 (1.1)	45.5 (1.1)	49.0 (1.1)	45.3 (1.1)
	Twenty	67.6 (1.0)	68.9 (1.0)	17.4 (0.8)	20.0 (0.9)	67.8 (1.0)	67.8 (1.0)	69.4 (1.0)	66.9 (1.1)
	Fifty	78.6 (0.9)	80.4 (0.9)	40.8 (1.1)	37.4 (1.1)	79.2 (0.9)	79.1 (0.9)	79.9 (0.9)	79.8 (0.9)

Table S8: Bias (MCSE), % Coverage (MCSE) and % Power (MCSE) for the scenario investigating the early treatment effect that ceases for the constant baseline hazard

		Hazard Ratio					ΔRMST		Time Ratio
	Effect time	Cox PH	PE1	PE2	LM	RP(PH)	RP(PH)	RP(TD)	Weibull AFT
Bias	Three	0.31 (0.003)	0.32 (0.003)	0.41 (0.005)	0.4 (0.005)	0.31 (0.003)	-3.7 (0.03)	-3.6 (0.03)	-0.32 (0.003)
	Ten	0.17 (0.003)	0.18 (0.003)	0.39 (0.005)	0.39 (0.005)	0.17 (0.003)	-2.3 (0.03)	-2.1 (0.03)	-0.19 (0.003)
	Twenty	0.07 (0.003)	0.08 (0.003)	0.18 (0.005)	0.16 (0.005)	0.07 (0.003)	-1.1 (0.03)	-1.0 (0.03)	-0.09 (0.003)
	Fifty	0.01 (0.003)	0.01 (0.003)	0.00 (0.005)	0.00 (0.005)	0.00 (0.003)	-0.2 (0.04)	-0.2 (0.04)	-0.01 (0.003)
Coverage	Three	39.5 (1.1)	39.2 (1.1)	57.4 (1.1)	58.6 (1.1)	39.7 (1.1)	21.0 (0.9)	22.5 (0.9)	33.7 (1.1)
	Ten	77.4 (0.9)	76.8 (0.9)	54.0 (1.1)	54.5 (1.1)	76.8 (0.9)	60.5 (1.1)	65.7 (1.1)	70.6 (1.0)
	Twenty	91.4 (0.6)	92.4 (0.6)	88.3 (0.7)	88.3 (0.7)	91.3 (0.6)	87.7 (0.7)	89.8 (0.7)	90.5 (0.7)
	Fifty	94.4 (0.5)	93.9 (0.5)	94.8 (0.5)	95.1 (0.5)	94.3 (0.5)	93.8 (0.5)	93.9 (0.5)	93.8 (0.5)
Power	Three	12.0 (0.7)	10.9 (0.7)	6.3 (0.5)	5.7 (0.5)	11.9 (0.7)	12.3 (0.7)	12.6 (0.7)	11.6 (0.7)
	Ten	36.8 (1.1)	35.9 (1.1)	5.7 (0.5)	5.6 (0.5)	36.4 (1.1)	36.8 (1.1)	41.7 (1.1)	35.3 (1.1)
	Twenty	66.3 (1.1)	65.4 (1.1)	18.3 (0.9)	21.3 (0.9)	66.8 (1.1)	66.4 (1.1)	68.8 (1.0)	65.7 (1.1)
	Fifty	79.8 (0.9)	80.0 (0.9)	44.7 (1.1)	43.6 (1.1)	80.1 (0.9)	80.2 (0.9)	79.8 (0.9)	80.3 (0.9)

Table S9: Bias (MCSE), % Coverage (MCSE) and % Power (MCSE) for the scenario investigating the early treatment effect that ceases for the increasing baseline hazard

		Hazard Ratio					ΔRMST		Time Ratio
	Effect time	Cox PH	PE1	PE2	LM	RP(PH)	RP(PH)	RP(TD)	Weibull AFT
Bias	Three	0.33 (0.003)	0.34 (0.003)	0.40 (0.005)	0.40 (0.005)	0.33 (0.003)	-3.9 (0.03)	-3.7 (0.03)	-0.34 (0.003)
	Ten	0.20 (0.003)	0.21 (0.003)	0.42 (0.004)	0.42 (0.005)	0.20 (0.003)	-3.2 (0.03)	-3.0 (0.03)	-0.27 (0.002)
	Twenty	0.08 (0.003)	0.10 (0.003)	0.19 (0.004)	0.17 (0.005)	0.08 (0.003)	-1.3 (0.03)	-1.1 (0.03)	-0.13 (0.003)
	Fifty	0.00 (0.003)	0.01 (0.003)	0.01 (0.005)	-0.01 (0.005)	0.00 (0.003)	-0.2 (0.03)	-0.2 (0.03)	-0.04 (0.003)
Coverage	Three	34.8 (1.1)	33.2 (1.1)	53.0 (1.1)	54.1 (1.1)	34.8 (1.1)	18.4 (0.9)	21.0 (0.9)	20.0 (0.9)
	Ten	71.1 (1.0)	69.2 (1.0)	47.5 (1.1)	47.9 (1.1)	71.0 (1.0)	27.5 (1.0)	31.6 (1.0)	27.2 (1.0)
	Twenty	89.1 (0.7)	89.8 (0.7)	86.3 (0.8)	85.2 (0.8)	89.2 (0.7)	83.2 (0.8)	86.2 (0.8)	81.1 (0.9)
	Fifty	95.3 (0.5)	95.6 (0.5)	95.9 (0.4)	95.1 (0.5)	95.2 (0.5)	94.8 (0.5)	94.7 (0.5)	94.3 (0.5)
Power	Three	7.9 (0.6)	7.1 (0.6)	4.6 (0.5)	5.5 (0.5)	8.1 (0.6)	8.1 (0.6)	8.9 (0.6)	8.5 (0.6)
	Ten	31.1 (1.0)	29.9 (1.0)	3.5 (0.4)	4.7 (0.5)	31.4 (1.0)	31.0 (1.0)	37.5 (1.1)	25.9 (1.0)
	Twenty	60.4 (1.1)	58.1 (1.1)	17.6 (0.9)	21.9 (0.9)	60.8 (1.1)	60.4 (1.1)	63.6 (1.1)	60.0 (1.1)
	Fifty	80.2 (0.9)	79.8 (0.9)	47.0 (1.1)	48.4 (1.1)	81.1 (0.9)	80.8 (0.9)	79.9 (0.9)	80.9 (0.9)

Power of the tests of equal survival curves

Supplementary Figure S5 presents the results of investigating the effect of non-constant hazard rates in the presence of an early effect that ceases for seven tests of equal survival functions. In the scenario equivalent to PH, only the LR and Cox tests achieve the power values anticipated under the design model, with the versatile test and the combined tests showing a small decrease in power. Under PH, all three FH tests (using early, middle and late weightings) had lower power than the expected 80%. The FH early test, with weighting emphasising earlier events in the survival curve, obtained the highest power when the treatment was only effective for short initial periods of 3% and 10% of study duration. The versatile test obtained the next highest power in the presence of the shorter effective periods but also had a power value closer to that observed for the LR and Cox tests when the treatment effect length was longer or persisted for the entire follow up. The RP(TD) combined test was closest to the versatile test, with allowing for a time-dependent treatment effect improving the power values slightly at each of the times investigated, relative to the RP(PH) combined test.

In general, the effect of non-constant event rates on the power of tests was consistent with what we observed for the modelling approaches. Decreases in the power loss were observed for a decreasing event rate compared to a constant event rate. An increasing event rate resulted in greater power losses than observed under a constant event rate. Whilst most changes in power observed attributable to a non-constant event rate were relatively modest for this simulation, depending on the test and the length of the effective period under consideration differences in power values ±5% were observed.

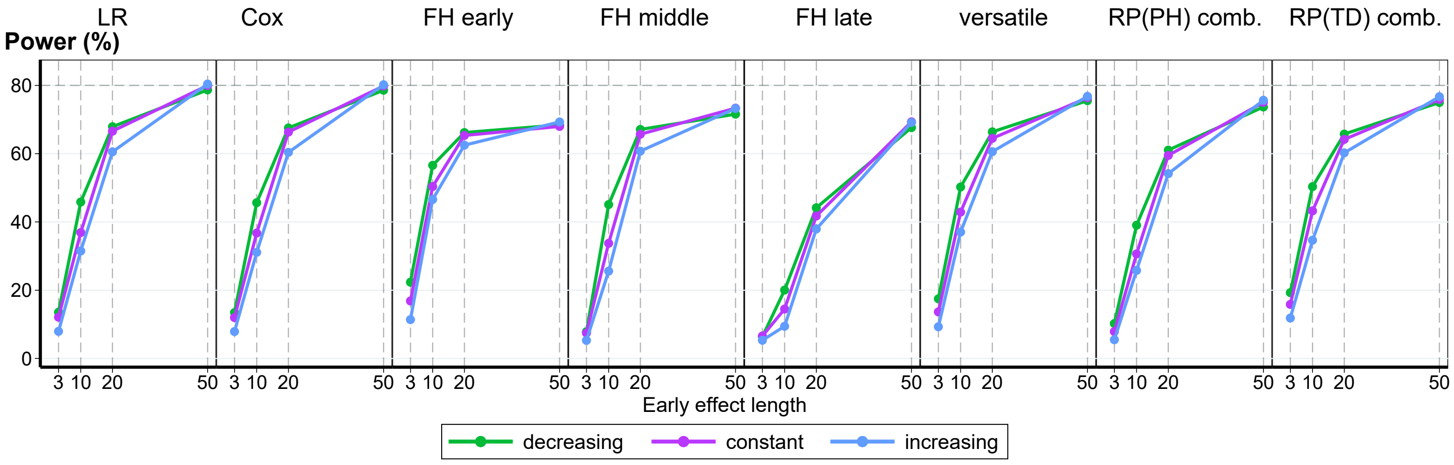

Supplementary Figure S5: Effect of non-constant event rates on the power of seven tests of equal survival function. The power of the z-test for the HR treatment effect from the Cox PH model is included in the panel as a comparator. The early effect period lengths investigated were $t_{early} = 3, 10, 20$ and 50 months, with the setting $t_{early} = 50$ representing PH.
