# Supplementary material for: Impact of a non-constant baseline hazard on detection of time-dependent treatment effects: a simulation study
Source: BMC Med Res Methodol. 2021 Aug 28;21:177. doi: 10.1186/s12874-021-01372-0 (PMC8399795; doi:10.1186/s12874-021-01372-0)
Supplement: Supplementary file 2 — Additional file 2. [file 12874_2021_1372_MOESM2_ESM.pdf]

```
/*	Simulations example: lag length

	Three hazard functions scenarios
	*constant	- lambdas(0.1) gammas(1.0)
	*decreasing	- lambdas(0.15) gammas(0.9)
	*increasing	- lambdas(0.07) gammas(1.1)

	Using n=202 for each run
	Decreasing lag to effect; lag time = 3
*/

version 15
set more off
clear

cd "`yourpath'"
*number of simulations
local sims = 100
local trt_eff1 = 0
local trt_eff2 = -0.4

*set seed for reproducibility
set seed 50621

*lag length
local laglen 3

*event rate scenario - decreasing
local lambda_1 0.15
local gamma_1 0.9
local evtype_1 "dec"

local timetot = 50
local time1 = `laglen'
local time2 = `timetot' - `time1'

clear
set obs 202
generate id = _n
*treatment variable, probability of 50% into each arm
generate trt = rbinomial(1,0.5)
*generate a time change
gen tchange1= cond(trt==1, `laglen', 500)

*user-defined hazard function
survsim survtime died, ///
	hazard( (`lambda_`rate'':*`gamma_`rate'':*#t:^(`gamma_`rate'':-1) ) :*	///
	( exp( (`trt_eff1':*trt):*(#t:<tchange1) :+		///
		(`trt_eff2':*trt):*(#t:>=tchange1) ) ) ) maxtime(50)  nodes(50)
```

```
*number of events pre and post lag time
count if died==1 & survtime <=`laglen'
local n_pre = r(N)
count if died==1 & survtime >`laglen' & survtime<=`timetot'
local n_post = r(N)
*by treatment group
count if died==1 & survtime <=`laglen' & trt==0
local n_pre_c = r(N)
count if died==1 & survtime <=`laglen' & trt==1
local n_pre_t = r(N)
count if died==1 & survtime >`laglen' & survtime<=`timetot' & trt==0
local n_post_c = r(N)
count if died==1 & survtime >`laglen' & survtime<=`timetot' & trt==1
local n_post_t = r(N)

stset survtime, failure(died = 1) id(id)

*Hazard Ratio from a Cox PH model
stcox trt, iterate(200)

*p-value from test of PH using Schoenfeld residuals
estat phtest

*Beta coefficients from a Cox PH model
stcox trt, iterate(200) nohr

*p-value from the logrank test and fleming-harrington tests
*equal weighting on all events
sts test trt, fh(0 0)

*early events weighting
sts test trt, fh(1 0)

*middle events weighting
sts test trt, fh(1 1)

*later events weighting
sts test trt, fh(0 1)


*versatile tests for equal, early or late (Karrison, 2016)
verswlr trt
local ver_std_p = r(pval)

*Cox model with time interaction for t>3 using stsplit
*includes landmark analyses

stsplit time_gt3, at(3)
*landmark analysis at t=3
```

```
*logrank test
sts test trt if time_gt3==3

stcox trt if time_gt3==3

*piecewise exponential model (at t=3)
*one estimate of treatment effect
streg trt ibn.time_gt3, dist(exponential) nocons


*two estimates of treatment effect
streg trt ibn.time_gt3 trt#ibn.time_gt3, dist(exponential) nocons

*estimate of treatment effect in interval time>3 (same as trt output)
lincom _b[_t:trt]  + _b[_t:1.trt#3.time_gt3], hr

*estimate of treatment effect when in interval time<=3
lincom  _b[_t:trt]  + _b[_t:1.trt#0.time_gt3], hr

stjoin

*Weibull shape and scale
streg trt, distribution(weibull) iterate(200)

*Time Ratio from accelerated failure time (AFT) model - Weibull
streg trt, distribution(weibull) time tr iterate(200)


*Estimate HR from RP model with 5 df - PH model parametric (ie no tvc option)
stpm2 trt, scale(hazard) df(5) failconvlininit eform iterate(200)

*Estimate diff in RMST from RP model with 5 df - PH model above
*using t* to be maximum uncensored event time
centile _t if _d==1, centile(100)
predictnl diff = ///
	predict(rmst at(trt 1) tmax(`t') ) - predict(rmst at(trt 0) tmax(`t') ), ///
	se(diff_se) p(diff_p) ci(diff_lci diff_uci)

*using t=50 to be the event time
predictnl diffm = ///
	predict(rmst at(trt 1) tmax(50) ) - predict(rmst at(trt 0) tmax(50) ), ///
	se(diffm_se) p(diffm_p) ci(diffm_lci diffm_uci)

*Royston-Parmar (RP) test for a generalized treatment effect (p-value)
*using default 5df (and as above) and specifying PH model
stctest rp trt, df(5) dftvc(0)

*Estimate HR from RP model with 5 df using dftvc(2) option
stpm2 trt, scale(hazard) df(5) tvc(trt) dftvc(2) failconvlininit eform
```

```
*Estimate diff in RMST from RP model with 5 df using tvc(2) option
*using t* to be maximum uncensored event time
centile _t if _d==1, centile(100)
local t = r(c_1)
predictnl diff_t = ///
	predict(rmst at(trt 1) tmax(`t') ) - predict(rmst at(trt 0) tmax(`t') ), ///
	 p(diff_p_t) ci(diff_lci_t diff_uci_t) se(diff_se_t)

*using t=50 to be the event time
predictnl diffm_t = ///
	predict(rmst at(trt 1) tmax(50) ) - predict(rmst at(trt 0) tmax(50) ), ///
	 p(diffm_p_t) ci(diffm_lci_t diffm_uci_t) se(diffm_se_t)


*Royston-Parmar (RP) test for a generalized treatment effect (p-value)
*using default 5df and using tvc(2) option
stctest rp trt, df(5) dftvc(2)

exit
```
